# Sex-related differences in hemato-biochemical indices of adult Vanaraja chickens during summer and winter seasons

**DOI:** 10.14202/vetworld.2017.176-180

**Published:** 2017-02-13

**Authors:** Kuldeep Kumar Panigrahy, Kumaresh Behera, Lal Mohan Mohapatra, Aditya Prasad Acharya, Kamdev Sethy, Sasmita Panda, Shailesh Kumar Gupta

**Affiliations:** 1 Division of Livestock Production and Management, ICAR - National Dairy Research Institute (NDRI), Karnal - 132 001, Haryana, India; 2 Department of Livestock Production and Management, College of Veterinary Sciences and Animal Husbandry, Bhubaneswar - 751 003, Odisha, India; 3 Department of Veterinary Pathology, College of Veterinary Sciences and Animal Husbandry, Bhubaneswar - 751 003, Odisha, India; 4 Department of Animal Nutrition, College of Veterinary Sciences and Animal Husbandry, Bhubaneswar - 751 003, Odisha, India

**Keywords:** indices, season, tropical climate, Vanaraja chicken, welfare

## Abstract

**Aim:**

The objective of this study was to evaluate the changes in hemato-biochemical indices in male and female Vanaraja chickens under tropical environment during summer and winter season.

**Materials and Methods:**

A total of 120 day-old sexed Vanaraja chicks were selected as experimental chickens and distributed equally in two groups having 60 female and 60 male chickens in each group, respectively. The experiment was continued for 8 weeks (56 days) and both male and female chickens were slaughtered by cervical dislocation method. All parameters were estimated at the end of the experiment in both seasons.

**Results:**

Male had higher blood glucose, Ca and P level. Blood glucose level significantly (p<0.05) reduced in summer. Female had higher total protein, albumin, globulin, and albumin/globulin ratio. Alanine aminotransferase and aspartate aminotransferase enzyme concentration were significantly (p<0.05) higher in summer. Total erythrocyte count, total leukocyte count, hemoglobin (Hb), Hb/lymphocyte ratio, and packed cell volume were significantly (p<0.05) higher in males. Mean corpuscular volume and mean corpuscular Hb were significantly (p<0.05) higher in females.

**Conclusion:**

Sex of chickens had a significant (p<0.05) effect on different parameters whereas season had nonsignificant (p>0.05) effect in most of the observed parameters. Hence, Vanaraja chickens are adaptable to local tropical climate and can be reared efficiently as backyard poultry.

## Introduction

Vanaraja is a multi colored synthetic backyard chicken developed by Project Directorate of Poultry Research, Hyderabad. Backyard poultry sector has major contribution toward strengthening of rural economy in India. Hence, it is necessary to study adaptability of these chickens by measuring the changes in different key indicators. Among all indices, hemato-biochemical parameters are promising indicators of physiological status and environment effect [1]. Hence, these indices are important for diagnosis and treatment of diseases and for measuring the environmental stress in chickens. The effect of season and sex on hemato-biochemical parameters were reported by many previous researchers [2,3]. Due to increased environmental temperature and humidity, there was marked reduction in performance of Vanarajachickens [4]. However, in some experiments, it was found that Vanaraja chickens are highly adapted to the local environment with intensive housing system [5]. In tropical climate indigenous chickens expressed low-stress condition than that of commercial strain [6].

Total protein, albumin and globulindecreases due to high environmental temperature. In Cobb broilers, total protein and albumin concentration decreases when ambient temperature increases above 30°C. Decreased plasma glucose concentration from 206 to 192 mg/dl was observed in broiler due to heat stress. Serum enzyme level is good indicator of physiological status of chickens. Different biochemical parameters - such as total protein, albumin, globulin, aspartate aminotransferase (AST), alanine aminotransferase (ALT), Ca and P - are also affected by sex of the chickens [2]. Higher level of total plasma protein was observed in females in comparison to males. Increased level of ALT and AST was observed in broiler chicken above 32°C temperature for 5 weeks of exposure. Significantly higher level of Ca and P was observed in female chicken [7]. Heat stress had significant effect on Ca and P level in chickens. Contrary to this, no significant effect ofheatwas observed in Ca level [8]. A significant effect of season was observed in Leghorn chickens and hemoglobin (Hb), packed cell volume (PCV), andtotal erythrocyte count (TEC) values changes during the heat stress in summer [9]. Sex-dependent variability was observed in blood hematological parameters such as PCV, TEC, total erythrocyte count (TLC), and different leukocytes cell counts in Indigenous chicken [2]. Contrary to this in local Saudi chicken, these parameters were found higher in male compared to female [10]. Most of the hematological parameters were higher in males than females [11]. Higher level of TEC, PCV, and Hb concentration was observed in males due to effect of androgen. Nonsignificant effect of age on mean corpuscular volume (MCV), mean corpuscular Hb (MCH), MCH concentration (MCHC), and differential leukocyte count was observed in local Saudi chicken [10]. Changes in MCHC and MCV between male and female were observed previously [11]. Changes in season also effect MCH and MCHC level were reported in previous experiments [8].

Findings on hemato-biochemical parameters by different Indian scientists [7,12] related to sex and season were available. There is little information about effect of season and sex on hemato-biochemical parameters of Vanaraja chickens. Hence, this experiment was conducted to investigate the effect of sex and season on hemato-biochemical parameters in Vanaraja chickens which will be helpful in evaluating the physiological and health status of the Vanaraja chickens.

## Materials and Methods

### Ethical approval

The research work was conducted in the Instructional Livestock Farm Complex, College of Veterinary Science and Animal Husbandry, Orissa University of Agriculture and Technology, Bhubaneswar, Odisha. The prior ethical approval from the Institutional Animal Ethics Committee, OUAT, College of Veterinary Science and Animal Husbandry, Bhubaneswar, Odisha-751003, was obtained for the use of animal in this study.

### Experimental design

A total of 120 Vanaraja chicks (60 male+60 female)were used for the experiment in each season. In each season, male and female chickens were divided into separate groups. The feeding and management of both groups were similar according to standard norms. Deep litter rearing arrangements were prepared 2 days before arrival of chicks. The deep litter house was divided into two compartments using wire netting. All chickens were marked using wing bands on 1^st^ day. Vaccination of chickens was done regularly according to standard guidelines. All chickens were fed as per BIS standard. The composition of dietis presented in Table-1.

**Table-1:** Ingredients (%) of broiler starter and finisher diets.

Components (% DM basis)	Pre-starter	Starter	Finisher
Dry matter	88.82^a^	89.16^a^	89.32^a^
Crude protein	22.92^a^	21.88^b^	19.65^c^
Metabolizable energy* (kcal/kg)	2970^a^	3092^b^	3166^c^
Ether extract	3.09^a^	3.56^a^	4.11^b^
Crude fiber	5.16^a^	4.82^b^	5.29^a^
Nitrogen free extract	64.91^a^	65.23^b^	66.32^c^
Acid insoluble ash	2.56^a^	2.52^a^	2.49^a^
Calcium	0.96^a^	0.95^a^	0.99^b^
Phosphorous	0.72^a^	0.71^a^	0.69^a^

Values bearing different superscripts within a row differed significantly (p<0.05)

The duration of experiments was for 8 weeks during winter (1^st^ December to 30^th^ January) and summer (1^st^ April to 30^th^ May). The experimental chickens were randomly divided into two groups,

1. Treatment- 1: Male Vanaraja – 60 chickens

2. Treatment- 2: Female Vanaraja – 60 chickens.

### Collection of blood sample and separation of serum

For hematological study, blood samples were collected aseptically at the end of theexperiment in summer and winter season of 30 selected chickens inethylenediaminetetraacetic acid containing vial. Blood samples were also collected for separation of serum and stored at −20°C for further use. These serum samples were used for biochemical studies. Hb was estimated using Sahli’shemoglobinometer; TEC using Neubauer chamber; other erythrocytes indices such as MCV, MCH, and MCHC were estimatedusing the standard formulae. The ALT and AST estimations were done after 24 h of collection. The serum biochemical parameters were estimated using kits AutoSpan Liquid Gold Diagnostic, India.

### Statistical analysis

The different hemato-biochemical parameters in male and female chickens were statistically analyzed using Statistical Package for Social Science version 17.0. Descriptive statistics in theform of mean ± standard error was used to measure all the parameters. Significance level was fixed at 5% level and p<0.05 were considered as significant.

## Results and Discussion

### Effect of sex and season on blood biochemical parameters

The biochemical parameters of male and female Vanaraja chickens during summer and winter are presented in Table-2. Male had significantly (p<0.05) higher blood glucose levelin both seasons than females. Contrary to this finding previous studies showed nonsignificant differences in glucose concentration between male and female [2,8,10]. This finding was opposite to finding in Aseel female chickens [7] where a higher level of glucose found in female chicken. Blood glucose level may increase with increase in temperature upto 305.95±3.23 mg/dl at 40-45°C in broiler chicken. In our study during summer season, the level of glucose was significantly (p<0.05) lower both in male and female than winter season. Previously increased blood glucose level was observed in chickens due to heat stress in summer [13]. This may be due to low feed intake during summer. No significant effectson blood glucose level observed in summer which is opposite tothe findings in previous study [13]. It may be due to negligible impact of heat in Vanaraja chickens in this study. Total protein, albumin and globulin concentration were significantly (p<0.05) higher in female in both season. The total protein level inthis study was similar to the finding of former study [13]. Similar to our result, increased total protein in female was observed in Aseel chickens [7], Nigerian local chicken [14], and Indigenous chickens [2]. This finding was contrary to the finding in Turkey under arid tropical environment [15]. Increased albumin concentration in females was similar to finding in Aseel chickens [7] and Nigerian local chicken [14]. This may be due to many physiological influences in female chickens. Significantly (p<0.05) high globulin concentration in female during both season is similar to finding in Turkey [15]. Nonsignificant (p>0.05) difference of total protein, albumin and globulin was observed in both season. Contrary to this, increased level of total protein was observed inthe previous experiment [13]. This may be due to increased heat stress on chickens during summer. No significant difference was observed in albumin concentration between winter and summer season. This finding was opposite to the observation in Ross strain broiler [8] and in Turkey under arid tropical environment [15]. Albumin/globulin (A/G) ratio was significantly higher in females in both seasons than males. There was nonsignificant (p>0.05) effect of season on A/G ratio but it slightly reduced in males. Nonsignificant (p>0.05) difference was observed in total cholesterol concentration between male and female in both seasons. This observation is similar to finding of Abdi-Hachesoo *et al*. [2]. Contrary to this, increased level of cholesterol was observed in female Assel chickens [7]. During summer, the cholesterol level was nonsignificantly higher in both male and females than winter. Increased level of cholesterol in summer was observed previously in many experiments. This finding supported a view of minor effect of summer stress in chickens. Nonsignificant (p>0.05) differences were observed in ALT and AST enzyme concentration between male and female in both seasons which is similar to finding of Abdi-Hachesoo *et al*. [2]. The ALT and AST enzyme concentration were significantly (p<0.05) higher in summer than winter. The serum concentration of Ca and P was significantly (p<0.05) higher in males in both seasons. Increased serum Ca and P in males in both seasons were similar to the finding of Abdi-Hachesoo *et al*. [2], Isidahomen *et al*. [14]. Contrary to this, higher level of Ca and P was observed in female Aseel chickens [7]. There was nonsignificant effect of season on Ca and P level. Contrary to this, increased P level was observed in Ross, Cobb and Hubbard broiler [8].

**Table-2:** Blood biochemical parameters of male and female Vanaraja chickens during summer and winter.

Parameters	Winter season		Summer season	
	
	Male	Female	Male	Female
Glucose (mg/dl)	210.36±8.35^aA^	179.89±5.67^bB^	186.34±7.39^cC^	156.67±4.89^dD^
Total protein (g/dl)	3.34±0.21^aA^	4.02±0.26^bB^	3.36±0.11^aA^	3.99±0.15^bB^
Albumin (g/dl)	1.98±0.03^aA^	2.57±0.06^bB^	1.95±0.05^aA^	2.50±0.07^bB^
Globulin (g/dl)	1.36±0.026^aA^	1.45±0.05^bB^	1.39±0.11^aA^	1.49±0.03^bB^
A/G ratio	1.45±0.06^aA^	1.77±0.03^bB^	1.40±0.08^aA^	1.67±0.04^bB^
Cholesterol (mg/dl)	182.35±0.30^aA^	181.21±0.32^aA^	186.11±0.24^aA^	184.19±0.26^aA^
ALT (IU/L)	27.29±1.29^aA^	26.86±1.12^aA^	46.38±1.36^bB^	43.55±1.98^bB^
AST (IU/L)	383.82±8.28^aA^	379.27±14.78^aA^	412.65±9.36^bB^	399.35±7.67^bB^
Calcium (mg/dl)	9.89±0.29^aA^	7.33±0.17^bB^	9.71±0.39^aA^	7.21±0.23^bB^
Phosphorus (mg/dl)	5.89±0.31^aA^	4.08±0.19^aA^	5.23±0.28^aA^	4.81±0.11^bB^

Values bearing different superscripts within a row in a single season differed significantly (p<0.05). A/G=Albumin/globulin, AST=Aspartate aminotransferase, ALT=Alanine aminotransferase

### Effect of sex and season on blood hematological parameters

The hematological parameters of male and female during summer and winter of Vanaraja chickens are presented in Table-3. Total TEC and Hb count were significantly (p<0.05) higher in males than females in both seasons. TEC and Hb concentration are lower than the finding in the previous study [12]. Higher level of TEC count in males is similar to the finding in Vanaraja chickens [12], colored broiler chickens [16], and Nigerian local chicken [14]. Higher level of Hb concentration in male Vanaraja chickens was also observed in the previous study [14]. Nonsignificant (p>0.05) differences were observed in TEC and Hb between summer and winter in both sexes. This finding is similar to the findings in Ross, Cobb, and Hubbard strain [8]. Contrary to this, higher level of TEC was observed during winter than summer season in Leghorn chickens at 40 weeks [9]. Decreased level of TEC was found due to high environment temperature stress. Significant effect of season on Hb concentration was observed in Ross and Cobb broiler [8] and Leghorn chickens at 40 weeks [9]. In both cases, Hb concentration increased due to heat stress for adaptation of chickens in changed environment. The total TLC count was significantly (p<0.05) higher in males than females in both seasons. In study of differential leukocyte counts (DLCs) (lymphocyte, heterophiles, monocytes, eosinophiles, and basophiles), no significant (p>0.05) difference was observed between male and female. TLC was lower than the finding in the previous study in Nicobari fowls [12]. Higher level of TLC count in males than females is similar to the report of Abdi-Hachesoo *et al*. [2], Albokhadaim *et al*. [10], and Isidahomen *et al*. [14]. The higher level of TLC in males may be due to the sexual hormone effect [2] or inherent sex difference. Earlier, no difference between different white blood cells between male and female with low value of TLC in females was observed in Sudanese indigenous chicken [17]. The percentage of sex-independent different leukocytes was also similar to the finding of Albokhadaim *et al*. [10]. Lymphocyte was a predominant TLC in both sexes. This is similar to the previous findings [2]. Nosignificant (p>0.05) effect of season was found in TLC and DLC. TLC was lower than the finding in the previous study in Nicobari fowls [12]. Higher level of TLC count in males than females is similar to earlier reports [2,10,14]. The higher level of TLC in males may be due to sex hormone effect [2] or inherent sex difference. Earlier, no difference between different white blood cells between male and female with low value of TLC in females was observed in Sudanese indigenous chicken [17]. The percentage of sex-independent different leukocytes was also similar to the previous findings [10]. Nonsignificant (p>0.05) differences were observed in Hb/lymphocyte ratio (H/L) ratio between male and female in both seasons. Contrary to our finding increased H/L ratio was observed in many experiments. H/L ratio may be increased from 0.39 to 0.56 in summer in broiler due to stress. Change in H/L ratio is a good indicator of stressful condition. PCV (%) was significantly (p<0.05) higher in males than females in both seasons. Higher values of PCV (%) in males were reported in many experiments [11,14]. The higher level of PCV reportedin males due to effect of androgens [2]. There was no significant (p>0.05) effect of season on PCV (%). Contrary to our finding, a decreased PCV (%) was observed in Cobb and Hubbard strains and in leghorn chickens [8]. MCV and MCH was significantly (p<0.05) higher in females in both season. Contrary to this finding higher value of MCV and MCHC was reported in male Nigerian local chicken [14]. No significant (p>0.05) differences were observed in MCHC between male and female. MCV and MCH were significantly (p<0.05) decreased in summer season in both sexes. Nonsignificant (p>0.05) MCHC value was observed between winter and summer. Higher level of MCHC in females and MCV in males and no difference in MCH between male and female was reported [11]. Contrary to our decreased MCV level in summer, previously many authors reported an increased level of MCV during summer due to heat stress. Further, MCH was decreased significantly (p<0.05) in summer season in both sexes. Decreased MCH was also reported in Ross and Cobb strains [8]. This may be due to nonsignificant (p>0.05) reduction in Hb concentration during summer. Nonsignificant (p>0.05) effect of season on MCHC were similar to the findings in Leghorn chickens [9] and Sudanese chicken ecotype [17]. Contrary to this report a low MCHC level was observed in Ross, Cobb and Hubbard strain [8].

**Table-3:** Blood hematological parameters of male and female Vanaraja chickens during summer and winter season.

Parameters	Winter		Summer	
	
	Male	Female	Male	Female
TEC (millions/mm^3^)	2.96±0.13^aA^	1.62±0.12^bB^	2.59±0.09^aA^	1.29±0.06^bB^
Hb (g/dl)	14.31±0.21^aA^	11.11±0.32^bB^	13.92±0.20^aA^	10.54±0.19^bB^
TLC (10^3^/µl)	63.59±0.65^aA^	49.81±0.98^bB^	62.38±0.51^aA^	48.98±0.73^bB^
Lymphocyte (%)	66.54±0.33^aA^	64.33±0.26^aA^	67.31±0.28^aA^	67.07±0.31^aA^
Heterophil (%)	26.72±0.16^aA^	27.88±0.26^aA^	26.67±0.22^aA^	25.87±0.21^aA^
Monocyte (%)	4.43±0.2^aA^	4.39±0.39^aA^	3.81±0.21^aA^	4.68±0.33^aA^
Eosinophil (%)	2.31±0.31^aA^	2.30±0.11^aA^	2.21±0.29^aA^	2.38±0.13^aA^
Basophil (%)	0	0	0	0
H/L ratio	0.40^aA^	0.43^aA^	0.39^aA^	0.38^aA^
PCV (%)	52.61±0.68^aA^	46.31±0.5^bB^	49.28±0.41^aA^	42.39±0.62^bB^
MCV (fl/cell)	138.54±4.89^aA^	142.54±5.41^bB^	114.31±6.48^aC^	118.54±6.31^bD^
MCH (pg/cell)	46.17±1.36^aA^	53.32±1.19^bB^	39.87±0.98^aC^	45.89±0.65^bD^
MCHC (g%)	35.61±0.65^aA^	36.03±0.98^aA^	36.53±0.51^aA^	36.98±0.73^aA^

Values bearing different superscripts within a row in a single season differed significantly (p<0.05). Hb=Hemoglobin, TEC=Total erythrocyte count, TLC=Total erythrocyte count, H/L=Hemoglobin/lymphocyte ratio, PCV=Packed cell volume, MCV=Mean corpuscular volume, MCH=Mean corpuscular hemoglobin, MCHC=Mean corpuscular hemoglobin concentration

## Conclusion

Among biochemical parameters, higher blood glucose, Ca and P level was observed in males. Blood glucose level significantly (p<0.05) reduced in summer. Female had higher total protein, albumin, globulin, and A/G ratio. ALT and AST enzyme concentration was significantly (p<0.05) higher in summer. Among hematological parameters, TEC, TLC, Hb, H/L ratio and PCV was significantly (p<0.05) higher in males. Lymphocyte was a predominant TLC in both sexes. MCV and MCH were significantly (p<0.05) higher in females. Effect of season was observed in MCV and MCH.

From this study, it is concluded that sex of chickens had a significant (p<0.05) effect on different parameters, whereas season had nonsignificant (p>0.05) effect in most of the observed parameters. Hence, Vanaraja chickens are adaptable to local tropical climate and can be reared as backyard poultry.

## Authors’ Contributions

KKP, KB, and LM designed the plan of work. APA, KKP, and KB performed laboratory investigation. KS and SP helped in the laboratory investigations. KKP, KB, KS, SP, and SKG participated in draftand revision of the manuscript. All authors read and approved the final manuscript.
